# Structural and optical characteristics of α-Bi_2_O_3_/ Bi_2_O_(3-x)_:Ho^3+^ thin films deposited by pulsed laser deposition for improved green and near-infrared emissions and photocatalytic activity

**DOI:** 10.1016/j.heliyon.2023.e23200

**Published:** 2023-12-03

**Authors:** J. Divya, N.J. Shivaramu, H.C. Swart

**Affiliations:** Department of Physics, University of the Free State, Bloemfontein, ZA-9300, South Africa

**Keywords:** Pulsed laser deposition, Oxygen pressures, Luminescence, Photocatalysis, α- Bi_2_O_3,_ Bi_2_O_2_._3_

## Abstract

Pulsed laser deposited films on glass substrate deposited at different substrate temperatures (T_s_) and partial pressures of oxygen, Ho^3+^-doped Bi_2_O_3_ films were produced. The degradation capability of the Rhodamine B dye using the Bi_2_O_3_:Ho^3+^ films was explored. The impact of the Bi_2_O_3_:Ho^3+^ content on the dye degradation performance was analyzed. The X-ray powder diffraction patterns showed that the films deposited at 400 °C had an α-Bi_2_O_3_ phase. The impacts of various T_s_ and O_2_ partial pressures were correlated with the surface morphology and the thickness of the films using results of field emission scanning electron microscope. The thin films deposited at a low O_2_ partial pressure of 5–20 mTorr at T_s_ = 400 °C exhibited nano-needles with an average size of 80 nm and a length of ∼750 nm. The estimated band gap of the prepared films was found to vary between 2.6 and 3.0 eV. The photoluminescence (PL) of the Bi_2_O_3_:Ho^3+^ thin films excited at 450 nm showed an intense green band emission observed at 548 nm, and the feeble emissions at 654 and 753 nm were ascribed to the transitions of Ho^3+^. The nano-needle particles of the α-Bi_2_O_3_:Ho^3+^ exhibited a maximum PL intensity for the 20 mTorr O_2_ partial pressure thin film. The films prepared in vacuum and with an O_2_ partial pressure of 5 mTorr exhibited a 41 % dye degradation efficiency during a duration of 270 min of the photocatalysis experiment.

## Highlights

• Surface characterization techniques complement each other.
• Good quality *Bi*_*2*_*O*_*3*_ PLD thin films.
• Highly efficient green luminescent thin films were obtained.
• Good photocatalytic effect on degradation of Rhodamine B Dye.

## Graphical abstract

**Figure:**
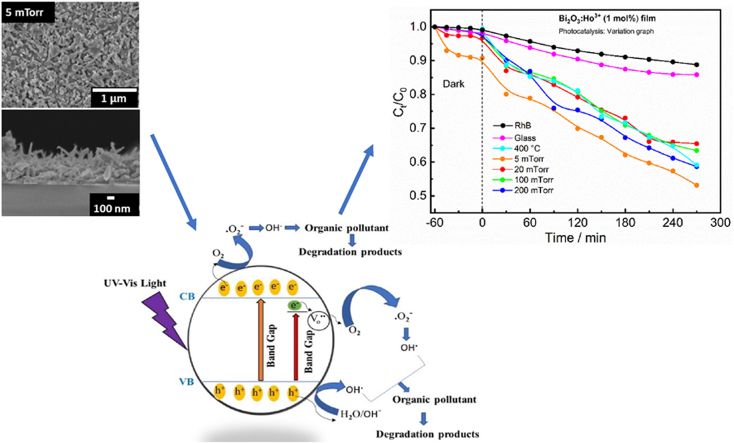

## 1 Introduction

In recent years, bismuth oxide (Bi_2_O_3_) thin films have gained wide interest owing to their good physical properties, which consist of a broad optical bandgap that varies from 2.0 to 3.9 eV, efficient photocatalytic activity, oxide ion conductivity, good photoluminescence (PL), high refractive index and dielectric permittivity [[1], [2], [3], [4]]. It has mainly four crystal polymorphisms such as α-, β-, γ- and δ-Bi_2_O_3_. Among all forms α-Bi_2_O_3_ is stable at room temperature (RT) and δ-Bi_2_O_3_ is stable at higher temperatures, and the remaining forms are metastable [5,6]. Bi_2_O_3_ thin films are widely studied and are involved in a multidirectional application, such as solar cells, gas, and humidity sensors, optical coatings, components in electronic circuitry, dye-sensitized photovoltaic cells (DSSCs) [7] and for heterogeneous photocatalysis [8,9]. Bi_2_O_3_ thin films were successfully grown by various methods namely spin-coating [9], radio-frequency magnetron sputtering [10], combination of a sol–gel process and electrospinning methods [7], thermal- and plasma-enhanced atomic layer deposition [11], photo-chemical solution deposition [12] and pulsed laser deposition (PLD) [6]. Paulo H.E. Falsetti et al. studied on the degradation of Rhodamine B (RhB) using β-Bi_2_O_3_ thin films under UV radiation and their results suggested that the thin films had good catalytic activity for the removal of RhB from water [9]. Juan C. Medina et al. showed the photocatalysis of δ-Bi_2_O_3_ thin films for the degradation of methyl orange (MO) under illumination of sunlight, white light, and UV radiation. The percentage of the photocatalytic degradation was considerably higher for UV radiation compared to sunlight and white light [13]. There are only limited reports on the photoluminescence of trivalent rare earth (RE^3+^) ion doped Bi_2_O_3_ thin films. Housei Akazawa, studied the PL properties of Er^3+^-doped Bi_2_O_3_ prepared by a sputtering technique on a SiO_2_ substrate. And his results showed very strong emission peaks at 1530 and 1560 nm, at a higher Er content (> 4 at%) [14]. He also investigated the band gap and PL of the α-Bi_2_O_3_ film under different deposition temperature and O_2_ flow rate [15]. The Er^3+^-doped Bi_2_O_3_ showed various crystal polymorphs, namely α, β, γ, δ-Bi_2_O_3_. However, the α-Bi_2_O_3_:Er^3+^ showed stronger PL intensities compared to the other polymorphs [16].

The study of the PL properties revealed that the RE-doped Bi_2_O_3_ displayed significantly higher luminescence intensity compared to the non-doped Bi_2_O_3_ [15]. Also, we expect that RE^3+^ sites can be easily substituted at Bi^3+^ sites because of similar ionic radii [1,3]. The ionic radii of Ho^3+^ ions (0.90 Å) are similar to the ionic radii of Bi^3+^ (1.03 Å) for coordination number six. And Ho^3+^ have higher luminescence from the visible to infrared regions, which belongs to their 4f–4f transitions [3]. The holmium element was rarely reported as a catalyst. There are a few reports on Ho^3+^ as a catalyst. Further, Ho^3+^ doping led to distortion in the host lattice, which reduces the electron hole recombination rate and generation of highly reactive hydroxyl radical, which helps to improve the photocatalysis process [3,17]. Therefore, in this study the structural, surface morphology, surface chemical, optical properties, and photocatalytic activity of Ho^3+^ activated Bi_2_O_3_ films deposited by PLD on glass substrate at different deposited substrate temperatures (T_s_) and oxygen partial pressures (PO_2_) were investigated. This study is unique in the sense that several surface characterization techniques in combination with optical measurements were used to explain the photocatalyst behaviour of Ho^3+^activated Bi_2_O_3_ films for degradation of RhB under UV–vis light.

## 2 Materials and methods

Bi_2_O_3_:Ho^3+^ (1 mol%) powder was synthesized by the co-precipitation technique [1]. The cleaned microscope glass substrates were positioned in the PLD chamber. The substrate temperature was maintained at RT, 200, and 400 °C during the deposition of the films in a vacuum (2.6 × 10^−5^ Torr). The films grown using the Nd:YAG laser (266 nm) and the target to substrate distance was fixed to 5 cm for all films. The time of deposition, laser energy and area of ablation were maintained at 15 min, 50 mJ and 1.5 mm^2^, respectively. The films were deposited with PO_2_ that varied between 5 and 200 mTorr, while the optimum substrate temperature of 400 °C was kept constant.

An Advance diffractometer (Bruker AXS D8) equipped with the CuK α source were performed to obtain the crystal structure information of the Bi_2_O_3_:Ho^3+^ PLD films. The surface morphologies, film thickness and elemental maps of the films were examined using a JEOL model JSM-7800 F field emission scanning electron microscopy (FESEM) (Tokyo, Japan) connected with an energy dispersive X-ray spectrometer (EDS). A Shimadzu atomic force microscopy (AFM) SPM-9600 model, a PHI 5400 XPS spectrometer and a PerkinElmer Lambda 950 UV–vis–NIR spectrophotometer were utilized for characterization. Complete information of the characterization methods can be obtained in Refs. [1,3]. The XPS spectra were fitted utilizing the XPS PEAK 4.1 programme. A FLS980 Edinburgh Instruments (Livingston, UK) was used to obtain PL spectra of the deposited films equipped with a 450 W Xe lamp.

The photocatalytic activity of the 1 mol% Ho^3+^ doped Bi_2_O_3_ films was evaluated for the RhB degradation in the UV–vis light at RT. The detail setup of the photocatalytic reactor was given in previous work [18]. The photocatalysis was carried out using a 100 mL of RhB solution with a 10 ppm dye concentration and a catalyst deposited film with a 2 × 2 cm^2^ size. The RhB solution was stirred for 60 min in the dark to attain an equilibrium. The suspensions were then transferred to a photoreactor, and the films were placed in the dye solution about 3 mm from the surface of the dye solution with the help of a stainless-steel wire. And irradiated with UV–vis light (350–800 nm) with constant stirring at a speed of 450 rpm for up to 270 min. During the irradiation, 3 mL samples were collected at 30 min intervals. The absorbance spectra of the dye were then collected with an UV–vis spectrophotometer.

## 3 Results and discussion

### 3.1 X-ray powder diffraction

**Fig. 1:**
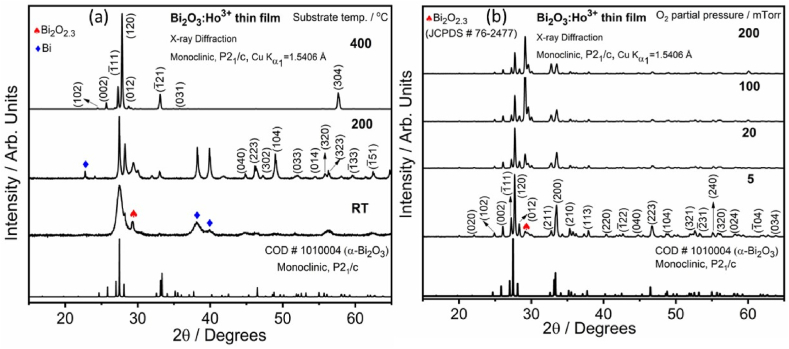
XRPD patterns of the Bi_2_O_3_:Ho^3+^ PLD films grown at the selected substrate temperatures (vacuum: 2.6 × 10^−5^ Torr) (a) and (b) O_2_ partial pressures (T_S_ = 400 °C).

As described in Fig. 1(a), the X-ray powder diffraction (XRPD) patterns of the Bi_2_O_3_:Ho^3+^ film grown at substrate temperatures ranging from RT-400 °C in vacuum (2.6 × 10^5^ Torr). The film deposited in vacuum at a substrate temperature of 400 °C displayed the Bragg reflections corresponding to the (102), (002), ($\bar{1}$ 11), (120), (012), ($\bar{1}$ 21), (031) and (304) planes of monoclinic Bi_2_O_3_ (COD #1010004) with a space group P2_1_/C as observed in the range of 15–65°. No other Bragg reflections, which could indicate the presence of any other crystallographic orientations of Bi_2_O_3_, Ho, or impurities phases, were detected. It was noticed that all the films have polycrystalline natures, and this indicates that single phase monoclinic Bi_2_O_3_ film were grown successfully on the glass, suggesting that Ho with 1 mol% doping has not altered the crystal structure of the Bi_2_O_3_ and will evenly replace the sites of the Bi^3+^ in the Bi_2_O_3_ matrix. However, considering the ionic radii for the 6-coordinated Bi^3+^ (1.03 Å) and the 6-coordinated Ho^3+^ (0.90 Å) [19] ions, there is a possibility that the Bi^3+^ ions be replaced by the Ho^3+^ ions during the doping process due to the same oxidation state and small ionic radii difference between the Bi^3+^ and Ho^3+^ ions. No secondary Ho phase was detected in the XRPD pattern due to low concentration of the Ho ions (1 mol%). In a previous report [3] on crystal structure of Bi_2_O_3_:Ho^3+^ powders there were also no secondary phases of Ho detected in the XRPD patterns aside from a small change in the unit cell volume of the crystal even dopant concentration of 5 mol% due to a slight variance in the ionic radii between Bi^3+^ and Ho^3+^ (6-coordinates). Additionally, the presence of Ho^3+^ in the Bi_2_O_3_ was confirmed by UV–Vis diffuse reflectance and PL results [3]. Further the confirmation of substitution of Bi^3+^ by Sm^3+^ (0.96 Å) ions in the Bi_2_O_3_ powders were confirmed by XPS results [20]. Similar XRPD patterns were recorded for the Bi_2_O_3_:Ho^3+^ film grown in the various PO_2_ between 5 and 200 mTorr (Fig. 1(b)). Due to an increase in PO_2_ the film with a preferential orientation was found to alter from the α-Bi_2_O_3_ (120) direction to non-stoichiometric Bi_2_O_2_._3_ (JCPDS Card 76–2477) of plane (107) in the PO_2_ range 5–200 mTorr. When the PO_2_ increases the intensity of the reflection corresponding to the (107) plane was increased. At low PO_2_, the difference in the orientation could be described by the reduction of the deposition rate due to rearrangement of atoms to obtain (107) reflection is low at a high deposition rate. The high O_2_ partial pressure (200 mTorr) induced a decreasing of the deposition rate due to collisions among the ejected species and the O_2_ molecules. This is due to the mean free path of oxygen molecules at high pressure is shorter than the target-substrate distance. Similarly growth rate dependence on PO_2_ has been noticed with other oxide materials [21,22]. The films deposited in the lower PO_2_ (5–20 mTorr) showed dominant α-Bi_2_O_3_ with small non-stoichiometric Bi_2_O_2_._3_ reflections. The films deposited at higher PO_2_ (100–200 mTorr) showed both α-Bi_2_O_3_ and non-stoichiometric Bi_2_O_2_._3_ phases and the non-stoichiometric Bi_2_O_2_._3_ phase was dominated. It indicated that the α-Bi_2_O_3_ phase was obtained at lower PO_2_ and the non-stoichiometric Bi_2_O_2_._3_ phase formed at higher PO_2_. The formation of the non-stoichiometric Bi_2_O_2_._3_ phase was since, a light O ablated atom was scattered with background oxygen molecules more strongly than the heavy Bi and Ho ablated atoms in the high O_2_ pressure. A relatively large number of oxygen therefore fails to arrive on the substrate surface, and this reductions of the O/Bi ratio of the film from its stoichiometric Bi_2_O_3_ [22,23].

The crystallite size (D) was determined from the XRPD data using the Scherrer formula. The reflection (120) of α-Bi_2_O_3_ was considered for the determination of the crystallite size and decrease with an increasing O_2_ partial pressure and its value varied in the range of 55–49 nm. While the D of the reflection (107) of the Bi_2_O_2_._3_ has increased between 22 and 44 nm. This can be ascribed to an increase mobility of the adatoms and coalescence of small crystallites to form bigger crystallites. From the XRPD analysis, the non-stoichiometric Bi_2_O_2_._3_ phase formation was influenced by the O_2_ partial pressures. It clearly indicates that the high PO_2_ induced the non-stoichiometric Bi_2_O_2_._3_ phase as more dominate than the α-Bi_2_O_3_.

### 3.2 Surface morphology and elemental analysis

**Fig. 2:**
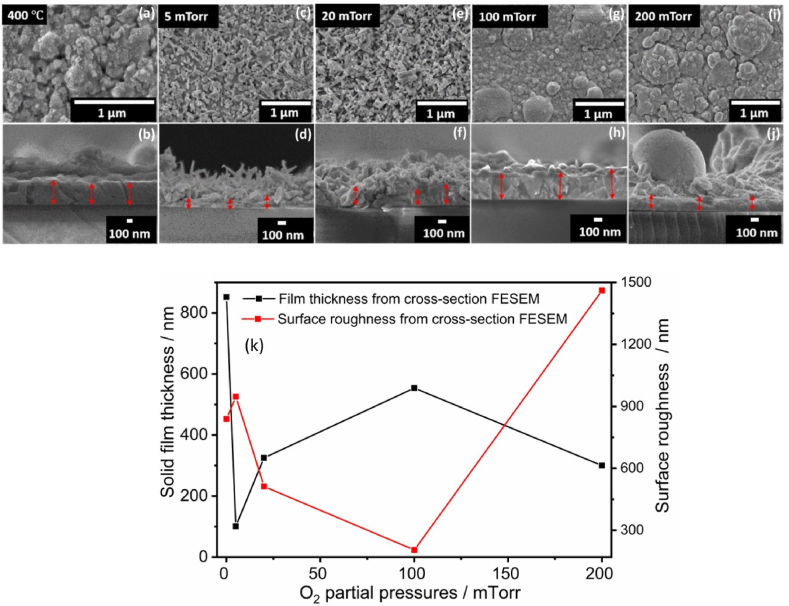
Top and cross-sectional views (FESEM) of Bi_2_O_3_:Ho^3+^ PLD films deposited at a substrate temperature of 400 °C (vacuum: 2.6 × 10^−5^ Torr) (a,b) and the selected PO_2_ (T_S_ = 400 °C) (*c*–j) (The measured solid film thickness were marked in the red arrow), and (k) the thickness of the solid part and uneven (rough) part of the thin films with different PO_2_.

**Fig. 3:**
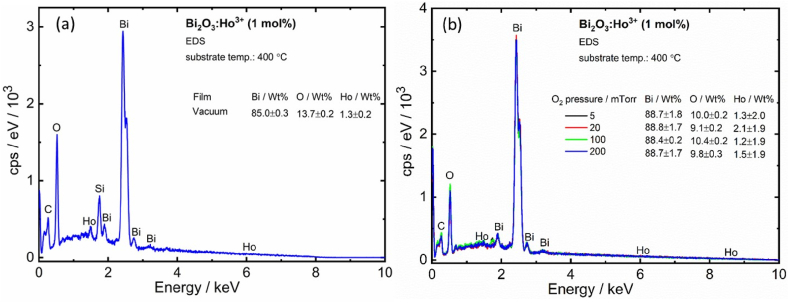
EDS spectra for Bi_2_O_3_:Ho^3+^ PLD films deposited in (a) vacuum and (b) PO_2_ of 5–200 mTorr.

**Fig. 4:**
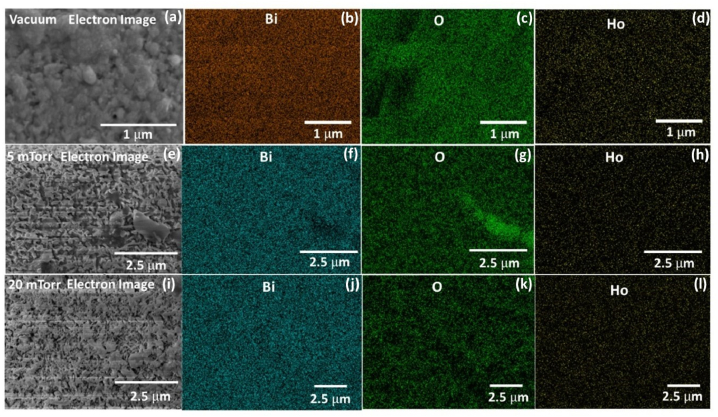
EDS elemental mapping images of Bi, O, Ho for PLD films deposited in vacuum (a–d) and PO_2_ of 5–20 mTorr (e–l).

In Fig. 2(a and b) the top and cross-sectional views of the PLD deposited films at a substrate temperature of 400 °C in vacuum (2.6 × 10^−5^ Torr) showed a good solid crystalline film with some features on the surface. The good crystallinity of the film is ascribed to the heat emerging during coalescence process at high temperatures. The higher substrate temperatures resulted in a higher mobility of the particles and hence resulting in the larger sized particles. This result good agreement with the XRPD patterns [24,25]. Fig. 2(c–j) also show SEM images and cross-sectional views of the PLD films surfaces deposited on the microscope glass at the selected PO_2_ between 5 and 200 mTorr at a Ts of 400 °C. At the lower PO_2_ (5 and 20 mTorr) the films showed a less dense needle like morphology. Specially, the 5 mTorr sample contained particles that have a lot of exposed surface area throughout the thin film. At the lower oxygen pressures (≤ 20 mTorr), the ablated species with a high kinetic energy avoid condensation because of the small possibility of these species collision with the O_2_ partial pressure gas species and thus reach to the substrate surface more easily to form the distinct grains [26,27]. The 20 mTorr thin film, however, is denser than the 5 mTorr film. This may lead to a higher photon absorption probability for the 20 mTorr film, but the 5 mTorr film may act as a better catalyst due to higher surface area available to react. The thin films deposited at low PO_2_ of 5–20 mTorr exhibited nano-needles with an average size of ∼80 nm and length of ∼750 nm. As the O_2_ partial pressure was increased the surface morphology changed with an increase in the particles size. Which led to the decrease of the kinetic energy, and the mean free path of the ejected species from the target owing to collisions between the ablated atoms and O_2_ at higher partial pressures during the film deposition. The ablated species with a lower kinetic energy that appeared at the substrate led to the reduction in surface diﬀusion. This condition favoured the grain growth and led to more dense grains with larger grain size due to agglomeration [26,27]. Please note: In contrast to the lower O_2_ partial pressure films, which only have a very thin solid component and instead consist of a less dense needle-like structure, the higher O_2_ partial pressure films all featured a clearly defined solid section with predicted features on top of the films. The thickness of the solid films varied from 100 to 850 nm and the thickness of the parts that are rougher compared to the solid films (surface roughness) approximated 205–1460 nm for the PLD deposited films, Fig. 2(k). As an example, the solid film thickness of the film deposited at 5 mTorr O_2_ partial pressure showed uneven behaviour due to the needle-like structure of the particles and its orientation in the different directions on the substrate, and as a result the solid boundaries are not clearly visible compared with the vacuum and high O_2_ partial pressure deposited films. Therefore, more deviation occurred in the indicated solid film thickness. The thickness of the solid film increased up to 100 mTorr O_2_ partial pressure and thereafter decreased at high O_2_ partial pressure (200 mTorr). The reduction of the solid thickness of the film at the high O_2_ partial pressure (200 mTorr) is ascribed to the decrease in the mean-free path of the vaporized species. A similar result was reported on ZnO:Eu films deposited by PLD [21]. The film deposited in vacuum exhibited high solid film thickness compared to the films deposited at O_2_ partial pressures, which might be due to a relatively large number of ablated atoms therefore more easily reach the substrate surface. The EDS spectra of Bi_2_O_3_:Ho^3+^ (1 mol%) films are depicted in Fig. 3 (a,b) and the apparent peaks in the EDS spectra indicate the presence of Bi, O, and Ho. Moreover, elemental mapping confirms the uniform distribution of the elements on the surface, as well as the homogeneous doping of Ho (Fig. 4(a–l)).

### 3.3 Atomic force microscopy analysis

**Fig. 5:**
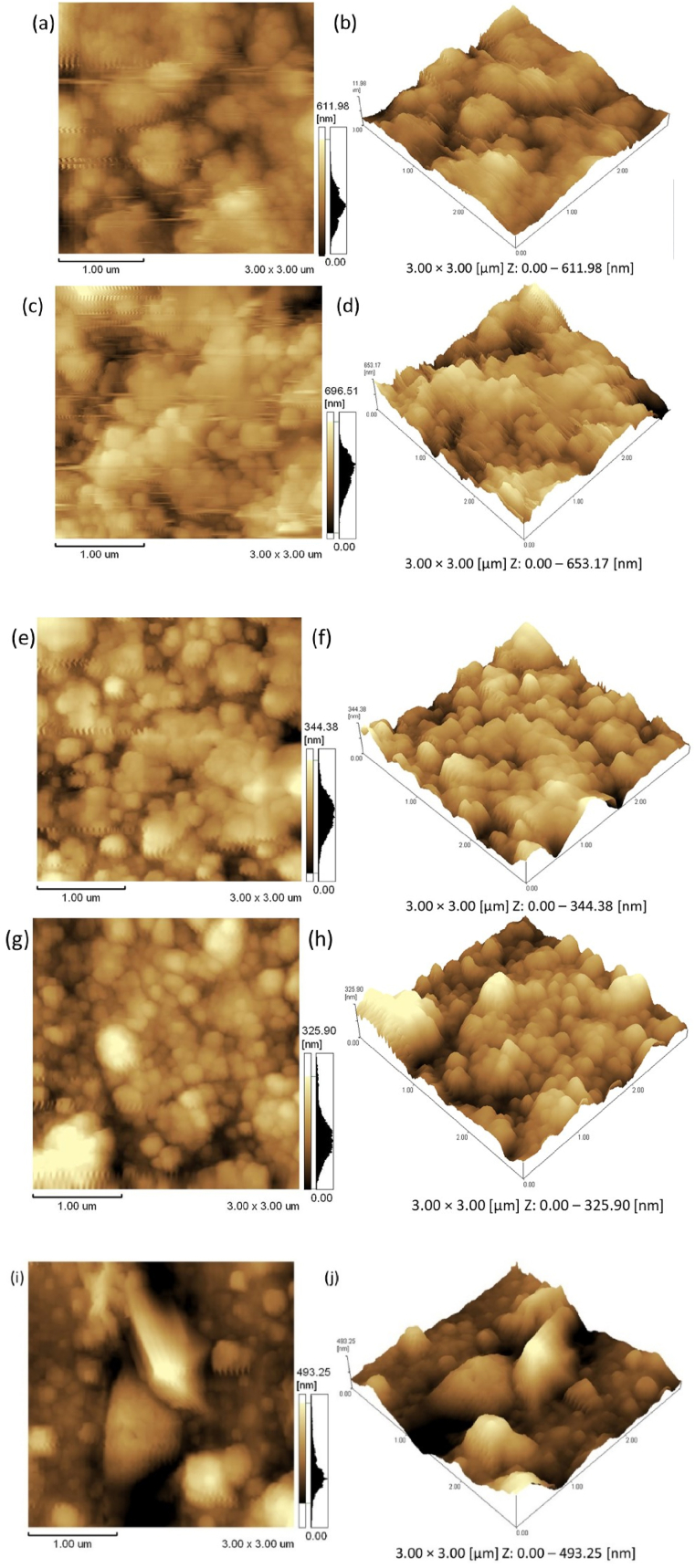
2D AFM images (a, c, e, g and i) and 3D AFM images (b, d, f, h and j) of Ho^3+^-doped Bi_2_O_3_ films. Where, (a,b): vacuum, (c,d): 5 mTorr, (e,f): 20 mTorr, (g,h): 100 mTorr and (i,j): 200 mTorr, respectively.

**Fig. 6:**
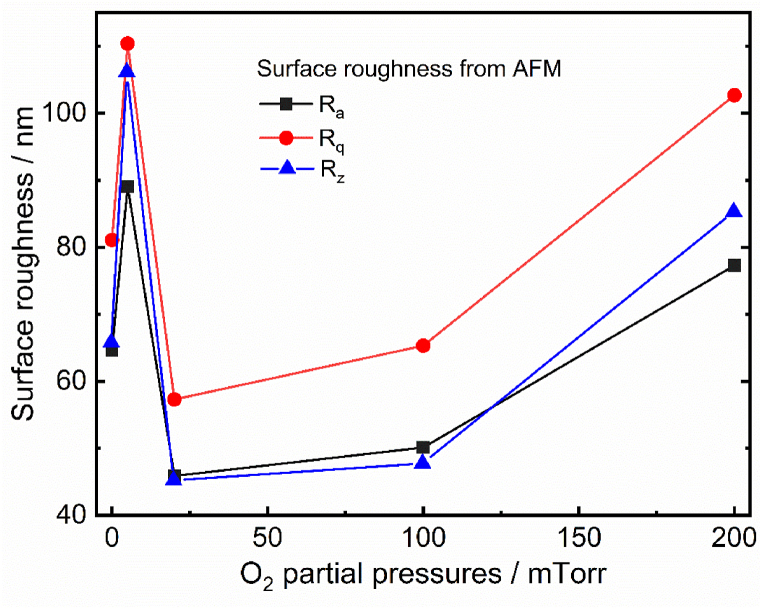
Variation graph of film surface roughness and thickness of the Bi_2_O_3_:Ho^3+^ (1 mol%) PLD films (0 mTorr on x-axis represents the vacuum film with pressure 2.6 × 10^−5^ Torr) as a function of PO_2_.

Fig. 5 (a−j) displays the two-dimensional (2D) and the three-dimensional (3D) AFM images of the Ho^3+^-doped Bi_2_O_3_ films deposited by the PLD method in vacuum and selected PO_2_ (5–200 mTorr). The AFM micrographs of the deposited films show non-uniform distribution of grains. The AFM micrographs additionally show that the particles were greatly unified with each other throughout the surface of the films. The film deposited in vacuum with substrate temperature of 400 °C revealed the particles were agglomerated. Less particles agglomeration of the deposited films was obtained at the low PO_2_ (5 and 20 mTorr) with the same substrate temperature. Increasing the PO_2_ from 100 to 200 mTorr led to more particle agglomeration, moreover the film deposited at the 100 mTorr O_2_ partial pressure revealed well defined spherical shape particles distributed at the film surface. The film surface roughness was analyzed by the arithmetic average roughness (R_a_), the root mean square (rms) roughness (R_q_) and ten-point average roughness (R_z._) The R_a_, rms and R_z_ roughness increased at 5 mTorr O_2_ partial pressure and decreased at 20 mTorr. Again, it further gradually increased with increasing O_2_ partial pressure in the range from 20 to 200 mTorr, which indicates the modified surface topography of the films. The surface topography can be tuned by increasing the O_2_ partial pressure during the film grown, which plays a vital role in the PL and photocatalytic activities. O_2_ partial pressure plays a very substantial role in the increase of the roughness of the film surfaces, which enhances the surface area and therefore increases the dye molecules adsorption, which in turn enhances the photocatalysis efficiency of the film. Fig. 6 confirmed that the film deposited at the 20 mTorr O_2_ partial pressure exhibited a smoother surface, while films deposited at high PO_2_ displayed a higher roughness due to the reflecting nucleation, coalescence and unceasing film growth processes. These results are comparable with those of Raoufi et al. [28,29]. It is clear that the AFM results cannot be used stand alone but must be used in conjuction with other techniques such as SEM in this case to obtain the full picture.

### 3.4 X-ray photoelectron spectroscopy study

**Fig. 7:**
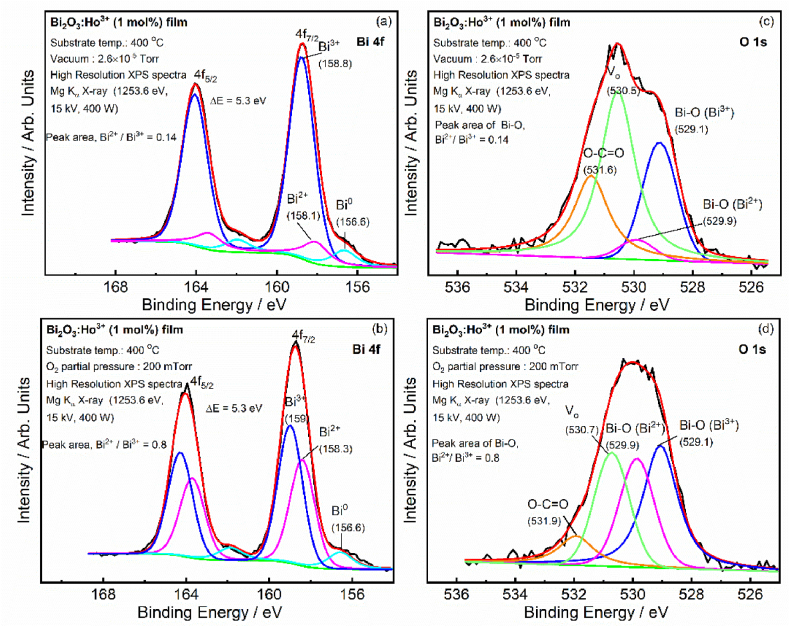
XPS of Bi 4f (a and b) and O 1s (c and d) for Bi_2_O_3_:Ho^3+^ PLD films grown in vacuum (2.6 × 10^−5^ Torr) and an O_2_ partial pressure of 200 mTorr at a T_S_ of 400 °C.

The deposited films were characterized via the Bi 4f and O 1s core levels as shown in Fig. 7. XPS fittings revealed a component at 156.6 eV, which clearly designated the presence of Bi in the metallic state (see Fig. 7(a and b)). A second component at higher binding energy (BE) (158.1/158.3 eV) has been assigned to Bi_2_O_2.3_-containing Bi^2+^ species, which occurred due to oxygen deficiencies in the films [18,30]. A third component at higher BE (158.8/159 eV) has been assigned to Bi_2_O_3_ – containing Bi^3+^ species [3,31]. The Bi 4f XPS region (Fig. 7 (a,b)) shows the doublet splitting, with the lower BE doublet assigned to Bi 4f_5/2_ and the higher BE, Bi 4f_3/2_ which is further confirmed by the spin–orbit coupling values of 5.3 eV for Bi 4f_5/2_ and Bi 4f_3/2_ [28]. The peaks area's ratio of the Bi^2+^ to Bi^3+^ of the film deposited at 200 mTorr O_2_ partial pressure is higher than the film grown in the vacuum in consistence with the XRPD patterns. The oxygen vacancies (V_o_) in the film can be confirmed using the O 1s core level XPS spectra [32]. The O 1s region was fitted with four components (Fig. 7(c and d)), the first component at low BE (529.1 eV) was assigned to the lattice oxygen (O_L_) in the Bi_2_O_3_. The second high BE component at 529.9 eV is ascribed to the O_L_ in the Bi_2_O_2.3_ (Bi^2+^). The third and fourth higher BE's components were assigned to V_o_ and surface impurities of hydroxide and *O*–C=O [1] respectively. The surface impurities occurred due to the exposure of the films to the air atmosphere prior of transferring them into the XPS vacuum chamber. The relative oxygen peak intensity of the oxygen in the Bi_2_O_2.3_ (Bi^2+^) increased at 200 mTorr. The film deposited under vacuum showed better stoichiometry (Bi_2_O_3_) than the film deposited under different O_2_ partial pressures.

### 3.5 UV–vis-DRS study

**Fig. 8:**
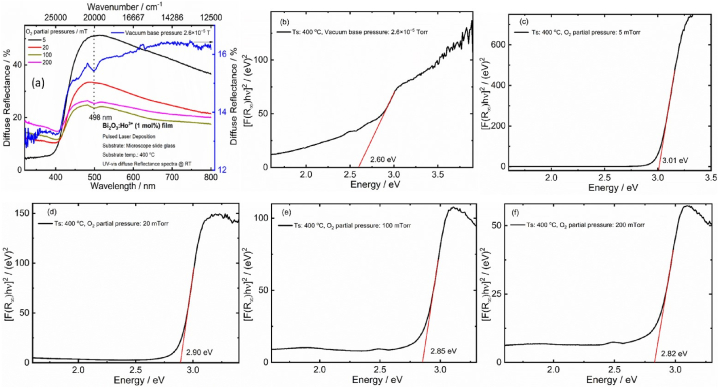
The diffuse reflectance spectra (a) and (b–f) (F (R_∞_)hv)^2^ was plotted with the energy of the Bi_2_O_3_:Ho^3+^ (1 mol%) PLD films.

The diffuse reflectance spectra (DRS) were measured for the Bi_2_O_3_:Ho^3+^ (1 mol%) films deposited by PLD are shown in Fig. 8(a). The spectrum displays an absorption edge at around 415 nm corresponding to the bandgap of the films. All the films begin to absorb radiation in the UV to near visible spectral range between 300 and 430 nm and were reflective in the visible spectral region, resulted in a light-yellow colour for the Bi_2_O_3_:Ho^3+^ films. An increase in the O_2_ partial pressure in the PLD chamber resulted in the reduction of the reflective intensity as displayed in Fig. 8(a). In addition, the film deposited in vacuum condition showed a low reflective intensity. It is clearly shown that a low O_2_ partial pressure amount was helpful to improve the reflectivity of the films. The DRS of the films deposited in vacuum and PO_2_ of 100 mTorr, 200 mTorr contained an absorb band centered at approximately 498 nm and can be ascribed to the structural defects. A similar result was observed in pervious results of Bi_2_O_3_:Sm^3+^, and Bi_2_O_3_:Ho^3+^ powder. The UV–vis diffuse reflectance (DR) spectrum of the Bi_2_O_3_:Ho^3+^ film mainly originated from direct transitions. Please note that the absorption of the 20 mTorr film is higher than that of the 5 mTorr film as speculated from the SEM images.

From the DR spectrum, the bandgap calculations were done by Kubelka–Munk (K-M) function. The reflectance spectrum of the film was converted to the K–M function [18]. The bandgap (E_g_) of the deposited films were determined from the plot of [F (R_∞_) hν]^2^ versus the photon energy (in eV) is extrapolated up to [F (R_∞_) hν]^2^ = 0 as presented in Fig. 8(b–f). The estimated value of E_g_ is 3.01 eV for the 5 mTorr O_2_ partial pressure. The optical bandgap decreased with the rise in O_2_ partial pressure; when the pressure was 20, 100 and 200 mTorr, the E_g_ was 2.90, 2.85, and 2.82 eV, respectively. Whereas the film deposited in vacuum show a value of E_g_ = 2.60 eV. The obtained optical bandgap are well consistent with our previous results [3,18] as well as others reports [11,15].

### 3.6 Photoluminescence

**Fig. 9:**
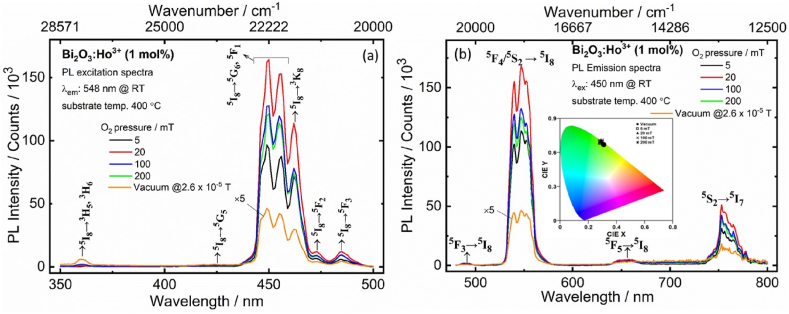
(a) The PLE and (b) PL spectra of the Bi_2_O_3_:Ho^3+^ (1 mol%) PLD films grown in vacuum and different PO_2_. Inset Fig. 5(b) illustration of the CIE Chromaticity coordinates.

Fig. 9(a and b) shows the PLE and PL spectra of the PLD films. Fig. 9(a) present the PLE spectra of the films recorded at an emission of 548 nm, and all the excited bands could be attributed to the intra-4f^10^ transitions of the Ho^3+^ [3,33]. All the excited bands derived from the ground ^5^I_8_ level to the excited state of the Ho^3+^. The strong intense excitation bands centered at 450 and 462 nm originated from the ^5^I_8_ →^5^G_6_, ^5^F_1_ and ^5^I_8_ →^3^K_8_, transitions, respectively. Similarly, relatively feeble excitation bands centered at 360 nm (^5^I_8_ →^3^H_5_, ^3^H_6_), 425 nm (^5^I_8_ →^5^G_5_), 473 nm (^5^I_8_ →^5^F_2_) and 485 nm (^5^I_8_ →^5^F_3_) [3,32]. From Fig. 9(a), the prominent intense PLE band (450 nm) has enhanced up to 20 mTorr of O_2_ partial pressure and then its intensity reduced.

For an excitation at 450 nm, the emission of the Bi_2_O_3_:Ho^3+^ film consisted of a prominent green emission from the ^5^F_4_/^5^S_2_ →^5^I_8_ transition at 548 nm and a near infrared (NIR) emission from ^5^F_2_ →^5^I_8_ at 753 nm, and an additional feeble blue emission from ^5^F_3_ →^5^I_8_ at 490 nm and a red emission from the ^5^F_5_ →^5^I_8_ transition of the Ho^3+^ centered at 655 nm (see Fig. 9(b)). To determine the optimal deposition conditions of the film, an increase of the O_2_ partial pressure the intensities of the green and NIR bands became stronger as a function of O_2_ partial pressure and reached a maximum at 20 mTorr. Which was probably due to the less dense needle like structures of the PLD films at the lower PO_2_. Smooth films may experience lower emission due to the internal reflections produced by smoother surfaces of the films [34] in the case of the solid more dense films at the higher O_2_ pressures. The luminescence quenching effect appeared with the O_2_ partial pressure over 20 mTorr, resulting in a decrease in the green and NIR emissions. Similar, results were observed in Sr_2_SiO_4_:Eu^3+^ thin film grown by PLD with different PO_2_ and reported by Jeong et al. [34]. The rms roughness and PL intensity of Sr_2_SiO_4_:Eu^3+^ films increased evenly with an increasing O_2_ pressure up to 150 mTorr and decreased evenly at higher O_2_ pressures 150–200 mTorr [34]. Furthermore, the Bi_2_O_2.3_ phase was stronger at higher deposited PO_2_ (> 20 mTorr). As a result, the Ho^3+^ ions occupied the distorted sites of the Bi^3+^ in the matrix, thus the probability of a non-radiative transition is stronger. The luminescence obtained from the film deposited in an O_2_ partial pressure condition is 10 times higher than the film deposited in vacuum due to aggregated particles in the vacuum. Indicating, that the films deposited in moderate PO_2_ are favourable for enhancing the luminescence of the Bi_2_O_3_:Ho^3+^ films [3,33].

**Fig. 10:**
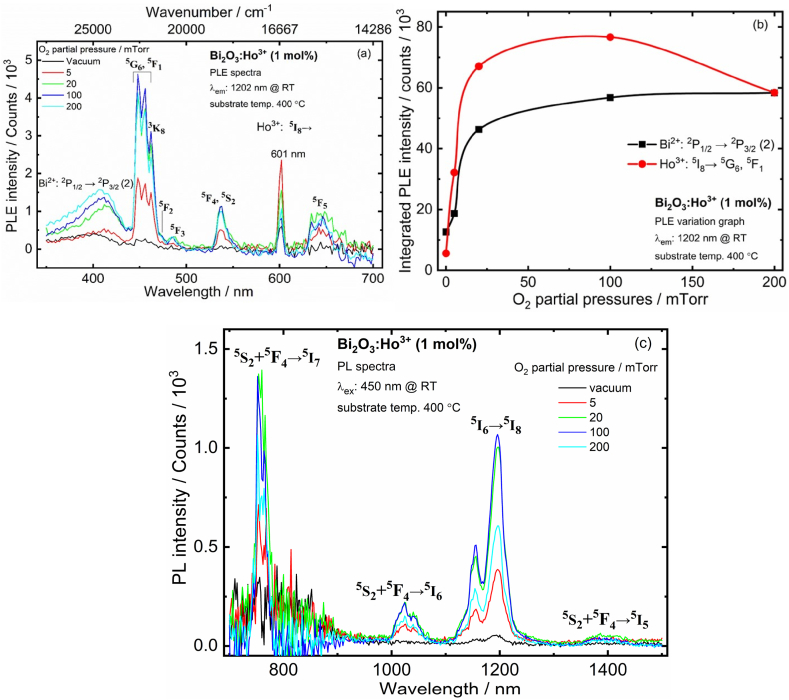
(a) The PLE spectra, (b) PLE intensity as a function of PO_2_ and (c) NIR-PL spectra of the Bi_2_O_3_:Ho^3+^ (1 mol%) PLD films grown in vacuum and different PO_2_ and films excited at 450 nm @ RT.

Fig. 10 (a) shows the PLE spectra of the Bi_2_O_3_:Ho^3+^ films obtained at an emission wavelength of 1202 nm. Several excitation bands were observed in the excitation spectrum with maxima at 448, 462, 473, 485, 536, and 644 nm, and these bands were assigned to the corresponding electronic transitions as ^5^I_8_ →^5^G_6_,^5^F_1_, ^5^I_8_ →^3^K_8_, ^5^I_8_ →^5^F_2_, ^5^I_8_ →^5^F_3_, ^5^I_8_ →^5^F_4_,^5^S_2_ and ^5^I_8_ →^5^F_5_ of Ho^3+^ ions, respectively. In addition, a broad band centered at 408 nm was observed, and this band was assigned to the Bi^2+^ transition as ^2^P_1/2_ →^2^P_3/2_ (2) from monitoring the NIR emission wavelength at 1202 nm, [35]. Liu et al. [31] found that Bi-doped BaBPO_5_ polycrystalline displayed broad excitation bands centered at 478 and 710 nm from monitoring the NIR emission wavelength at 1164 nm and these bands were assigned to the Bi^2+^ [32]. Other reports showed that the Bi-doped SrAl_4_O_7_ phosphor displayed four excitation bands centered at ∼277, 325, 416, and 590 nm if monitored at 710 nm. Three of them, positioned at 277, 416, and 590 nm were correspond to the transitions from ground ^2^P_1/2_ level to the ^2^S_1/2_, ^2^P_3/2_ (2) and ^2^P_3/2_ (1) of the Bi^2+^ ions, respectively [36]. Furthermore, an excitation peak was observed at 601 nm for all films due to the second harmonic oscillations.

The integrated PLE intensity of the prominent Ho^3+^: ^5^I_8_ →^5^G_6_,^5^F_1_ transition has increased up to the 100 mTorr O_2_ partial pressure and thereafter decreased (Fig. 10 (b)). Moreover, the integrated PLE intensity of Bi^2+^: ^2^P_1/2_ →^2^P_3/2_ (2) has exponentially grown with O_2_ partial pressure and is shown in Fig. 10 (b). The PL intensity of the Ho^3+^ doped Bi_2_O_3_ films has been monitored on excitation with 450 nm in the 700–1500 nm region, and the spectra thus obtained are shown in Fig. 10 (c). The emission spectra contain several emission peaks centered at 753, 1023, 1202, and 1385 nm and these peaks were assigned to arise due to ^5^S_2_+^5^F_4_ →^5^I_7_, ^5^S_2_+^5^F_4_ →^5^I_6_, ^5^I_6_ →^5^I_8_ and ^5^S_2_+^5^F_4_ →^5^I_5_, transitions, respectively [37,38]. The variation in PL intensities of all NIR emissions with PO_2_ reflected the same as the PLE spectra. The decreased PL intensities after 100 mTorr were due to changes in the local symmetry around Ho^3+^ [35,37,38].

### 3.7 Photoluminescence lifetimes

**Fig. 11:**
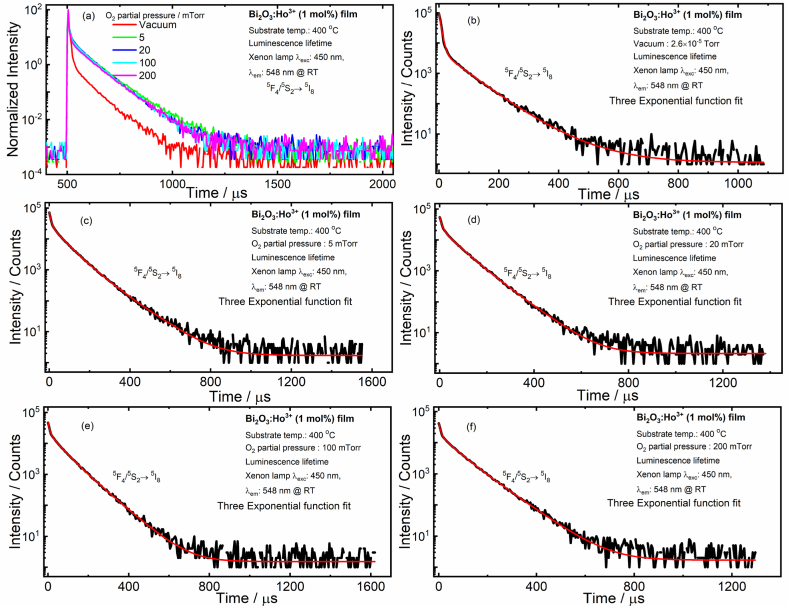
PL lifetime decay curves (a) and (b–f) fitted decay curves for the PLD films deposited in vacuum and different PO_2_ and films excited at 450 nm and measured at 548 nm @ RT.

It is important to know the recombination processes of charge carriers in trap defect states. The quality of deposited Bi_2_O_3_:Ho^3+^ films and their role in photocatalytic performance have been analyzed by estimating the activator and defect lifetimes using PL lifetime spectroscopy. PL lifetime decay curves were measured for the most intense transition of the green emission from the ^5^F_4_/^5^S_2_ →^5^I_8_ level of Ho^3+^ with an excitation of 450 nm. The PL lifetime decay for the emission at 548 nm, with an excitation of 450 nm in the doped Bi_2_O_3_ films deposited in vacuum and selected PO_2_ (5–200 mTorr), at the optimized substrate temperature (400 °C) is shown in Fig. 11 (a), respectively. The lifetime decay in all deposited films was fitted with a three-exponential function. The summary of the PL lifetime data of Ho^3+^ doped Bi_2_O_3_ films at the 450 nm excitation wavelength is displayed in Fig. 11(b–f).

**Fig. 12:**
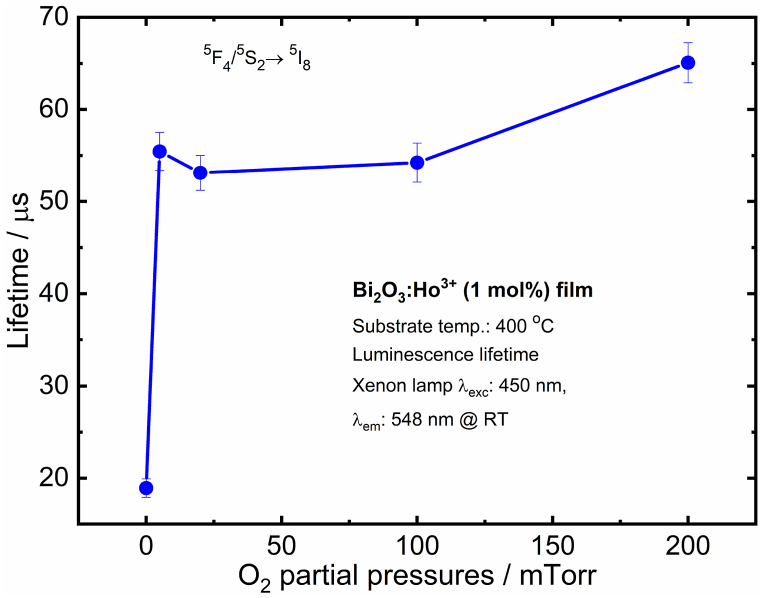
The average PL lifetime of the Bi_2_O_3_:Ho^3+^ PLD films (0 mTorr on x-axis represents vacuum with a pressure 2.6 × 10^−5^ Torr) with different PO_2_.

The tri-exponential fits that yielded the different decay times are summarized in Table 1. For the Bi_2_O_3_:Ho^3+^ vacuum films, the decay is based on three decay times: 7 ± 1 μs fast decay, 57 ± 2 μs a modest decay and 115 ± 20 μs a slow decay [38]. The fast decay time (τ_1_), for the Bi_2_O_3_:Ho^3+^ films is ascribed to Ho^3+^ ions close to the surface [39] and its decay time has not altered a lot in all the deposited films. The modest decay time (τ_2_) is attributed to Ho^3+^ ions in the bulk. The major contributing was from the slow decaying component (τ_3_) that might arise from defects. Both τ_2_ and τ_3_ have decreased in the films deposited in the PO_2_ compared with the vacuum deposited film, which was probably due to a combination effect of the Ho^3+^ ions occupying the stoichiometric Bi_2_O_3_ and non-stoichiometric Bi_2_O_2.3_, V_o_, and surface morphologies. As seen from Fig. 12, the average lifetime was shorter in the vacuum as compared to those of the different O_2_ partial pressures. An optimal oxygen vacancy formation in the Bi_2_O_3_ host might facilitate the energy transfer and relaxation processes better.

**Table 1:** PL lifetime in microseconds for the ^5^F_4_/^5^S_2_→^5^I_8_, Ho^3+^ transition of the Bi_2_O_3_:Ho^3+^ PLD films.

PO_2_	$\tau_{1}$/μ s	$\tau_{2}$/μ s	$\tau_{3}$/μ s	$\tau_{\text{ave}}$/μ s
Vacuum	7 ± 0.03	57 ± 2	115 ± 20	19 ± 1
5 mTorr	8 ± 0.2	49 ± 2	89 ± 2	55 ± 2
20 mTorr	8 ± 0.2	45 ± 2	78 ± 2	53 ± 2
100 mTorr	8 ± 0.2	47 ± 2	80 ± 2	54 ± 2
200 mTorr	6 ± 0.5	43 ± 3	80 ± 1	65 ± 2

### 3.8 Photocatalysis

**Fig. 13:**
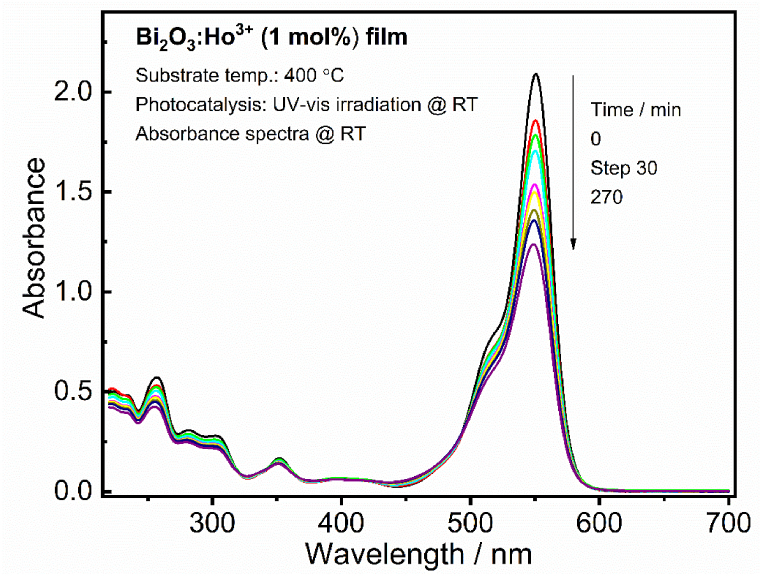
Absorbance spectra of the RhB in the presence of the Bi_2_O_3_:Ho^3+^ (1 mol%) PLD film grown in vacuum (2.6 × 10^−5^ Torr) at T_S_ of 400 °C under different UV–vis light exposure.

To know the relationship between the photocatalytic property of the PLD films deposited at 400 °C (vacuum: 2.6 × 10^−5^ Torr) and the different PO_2_ 5, 20, 100 and 200 mTorr (T_s_ = 400 °C) the photocatalytic activity of the Bi_2_O_3_:Ho^3+^ (1 mol%) films has been examined by selecting the degradation of RhB. Earlier to the UV–vis light irradiation adsorption-desorption measurement was done in the dark for 60 min and the photolysis of RhB (without catalyst) and with catalysis in the presence of a microscope glass was done and the results are tabulated in Table 2. Fig. 13 Shows the photocatalytic absorbance spectra of the film deposited at a T_s_ of 400 °C in vacuum (2.6 × 10^−5^ Torr) (size 2×2cm) from 0 to 270 min (with time intervals of 30 min), under the UV–vis light irradiation. These results display the maximum intensity of the RhB absorption spectra at 552 nm which decreased with an increase in the UV–vis light irradiation time. It represents the destruction of the RhB and the formation of some intermediates [1].

**Table 2:** The concentrations and fractions of the RhB adsorbed in dark of the Bi_2_O_3_:Ho^3+^ (1 mol%) films.

Samples	Dye sol. (ppm)	C_ads_= (C_t_/C_0_)	% θ_ads_ = [(1− C_ads_) × 100]	Dye_adsorbed_ (ppm)	C_1_ (ppm)
Glass	10	0.98	2	0.2	9.8
Vacuum	10	0.99	1	0.1	9.9
5 mTorr	10	0.90	10	1.0	9.0
20 mTorr	10	0.96	4	0.4	9.6
100 mTorr 200 mTorr	10 10	0.98 0.97	2 3	0.2 0.3	9.8 9.7

**Fig. 14:**
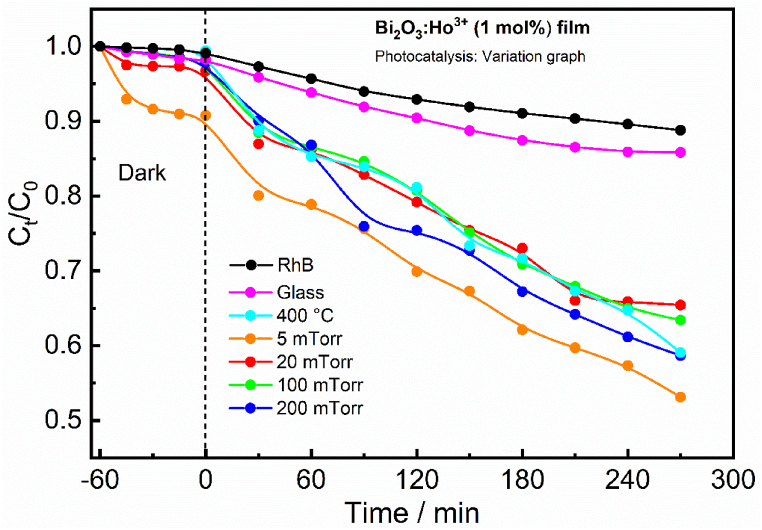
The variation of the RhB degradation in the presence of the Bi_2_O_3_:Ho^3+^ (1 mol%) PLD films at different UV–vis light exposure.

Fig. 14 presents the changes in the RhB concentration due to the UV–vis light irradiation in the presence of the different thin film catalysts. Where C_t_ and C_o_, in Fig. 14 are the reaction concentration of the RhB at different times and the initial concentration of the RhB after the equilibrium adsorption. All the Bi_2_O_3_:Ho^3+^ (1 mol%) films showed improved photocatalytic activity in comparison with the photolysis of RhB, without a catalyst. Which indicated that the Bi_2_O_3_:Ho^3+^ (1 mol%) films acted as an efficient catalyst for the degradation of the RhB. And the order of degradation efficiency of the RhB for the different samples could be as follows: 5 mTorr (41 %), vacuum (41 %), 200 mTorr (40 %), 100 mTorr (35 %) and 20 mTorr (33 %). The degradation efficiency % θ_deg_ was determined by using the formula below (1):

(1) $$\;\%\theta_{\deg}=\left[(1‐C_{\deg})\times 100\right]$$

Where, C_deg_ = C_t_/C_o_, Co is the concentration of the RhB at t = 0 after the equilibrium adsorption and C_t_ the reaction concentration of the RhB after different time intervals (t).

**Fig. 15:**
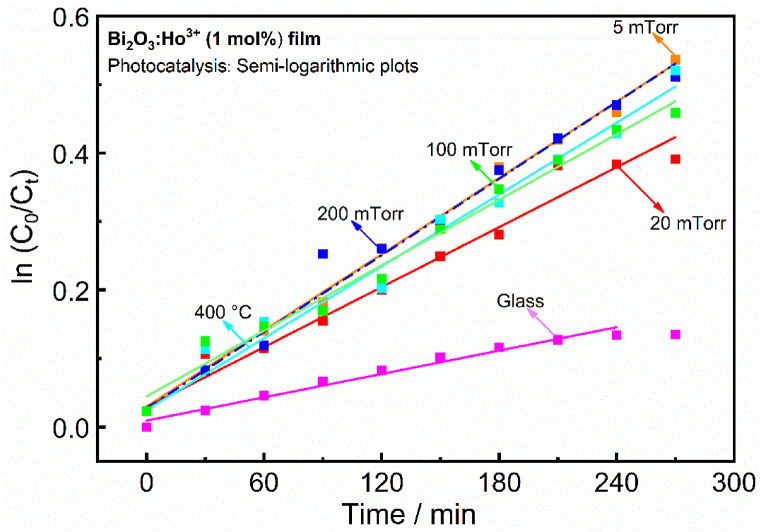
Plots of ln (C_0_/C_t_) vs irradiation time for the degradation of RhB in the presence of Bi_2_O_3_:Ho^3+^ (1 mol%) films under UV–vis light exposure.

A clear relationship between the surface morphology and the photocatalytic activity was observed for the PLD Bi_2_O_3_:Ho^3+^ (1 mol%) films. The best photocatalytic activity and adsorption for the RhB was obtained for the film deposited at 5 mTorr (41 %), and was attributed to its surface morphology (see FESEM image and cross section of the 5 mTorr film as shown in Fig. 2). The film contained needle shape particles all-over the film surface, and supported a higher photocatalytic activity as compared to the other Bi_2_O_3_:Ho^3+^ (1 mol%) films [9]. AFM study helped to show that there is a relation between the photodegrading process and the surface roughness, the films deposited at 5 mTorr and 200 mTorr have higher surface roughness that led to high surface area and hence high adsorption and photocatalytic activity. The photocatalytic activity increased with an increase in surface roughness [40]. Fig. 15 clearly shows that the rate constants (k) for the Bi_2_O_3_:Ho^3+^ (1 mol%) films under UV–vis light followed a pseudo-first-order reaction and the obtained rate constant values of the various Bi_2_O_3_:Ho^3+^ (1 mol%) films are tabulated in Table 3 [1].

**Table 3:** Parameters of photocatalytic activity in degradation of the RhB by Bi_2_O_3_:Ho^3+^ (1 mol%) films under UV–visible irradiation.

Samples	C_1_ (ppm)	Photodegradation efficiency, η (%)	Rate constants, k × 10^−5^ (min^−1^)	Correlation constants, R^2^	Dye_deg_ (ppm)
RhB	10	11	–	–	1.10
Glass	9.8	13	57 ± 3	0.98	1.27
Vacuum	9.9	41	175 ± 9	0.98	4.06
5 mTorr 20 mTorr	9.0 9.6	41 33	186 ± 7 146 ± 9	0.99 0.96	3.69 3.17
100 mTorr 200 mTorr	9.8 10	35 40	160 ± 7 186 ± 9	0.98 0.98	3.43 3.88

### 3.9 The proposed mechanism for the photocatalytic activity of RhB

**Fig. 16:**
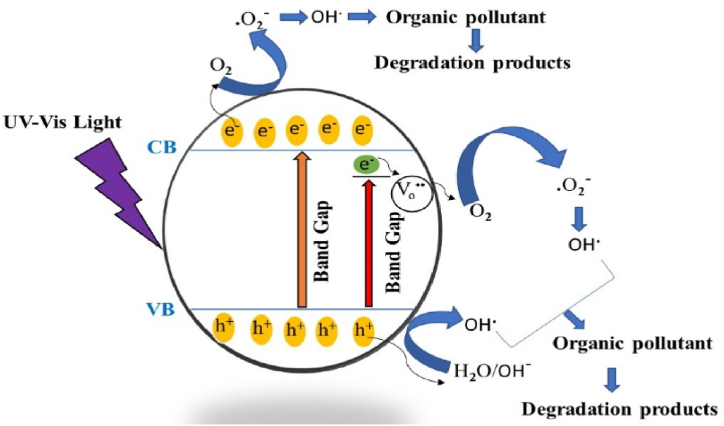
Mechanism of photocatalytic RhB dye degradation.

When a photon with energy of hν (UV–vis irradiation) is incident on the film surface, the photons with their energy equal to or bigger than the band gap of the film is absorbed by the Bi_2_O_3_:Ho^3+^ (1 mol%). This leads to the generation of electrons ($e_{\text{CB}}^{-}$) and holes $\;(h_{\text{VB}}^{+}$) at the conduction band (CB) and the valence band (VB) (Eq. (2)), respectively. These photon-generated $h_{\text{VB}}^{+}$ act as oxidation sites and contribute to the generation of hydroxyl radicals (OH^•^) (Eqs. (3), (4)). The $e_{\text{CB}}^{-}$ acts as reduction sites, which react with the surface oxygen (O_2_) that leads to the generation of superoxide anion radicles (O_2_^•-^) (Eq. (5))**.** Additionally, the presence of oxygen vacancies (V_o_^••^) help to increase the surface OH groups. The V_o_^••^ might absorb more O_2_ reacting with the captured electrons (Eqs. (6), (7)). Or V_o_^••^ may become traps for the ejected electrons (Eqs. (8), (9)), which can control the photo-induced electrons and holes recombination that leads to the better catalytic activity of the RhB and the schematic representation is given in Fig. 16. Both OH^•^ and O_2_^•-^serve as an oxidant to degrade the organic pollutant [10] (Eq. (10)). The reactions are given below [1,9,10]:

(2) $$h\nu +\alpha ‐{\text{Bi}}_{2}O_{3}:{\text{Ho}}^{3+}\text{film}\to \alpha ‐{\text{Bi}}_{2}O_{3}:{\text{Ho}}^{3+}\;\left(e_{\text{CB}}^{‐}+h_{\text{VB}}^{+}\right)$$

(3) $$h_{\text{VB}}^{+}+H_{2}O\;\to \;{\text{OH}}^{•}+H^{+}\;\text{or}$$

(4) $$h_{\text{VB}}^{+}+{\text{OH}}^{\;–}\;\to \;{\text{OH}}^{•}$$

(5) $$e_{\text{CB}}^{-}+O_{2}\;\to \;O_{2}^{•\;–}$$

or

(6) $$V_{o}^{••}+O_{2}\;\to \;\text{absorbed}\;\text{oxygen}\;\left(O_{2}^{\;–}\;/\;O_{2}^{2‐}\;/O^{\;–}\;\right)$$

(7) $$\left(O_{2}^{\;–}\;/\;O_{2}^{2‐}\;/O^{\;–}\;\right)+e^{\;–}\;\to \;O_{2}^{•}$$

or

(8) $$V_{o}^{••}{+e}^{–}\;\to V_{o}^{•}$$

(9) $$V_{o}^{•}+O_{2}\to \;V_{o}^{••}+O_{2}^{•\;–}$$

(10) $$\text{RhB}+O_{2}^{•\;–}\;/\;{\text{OH}}^{•}\;/\;O_{2}^{•}\to \;\text{degradation}\;\text{product}$$

where, oxygen vacancies are represented by (V_o_^••^), an electron trapped at oxygen vacancy is represented by V_o_^•^ [41,42].

## 4 Conclusions

α-Bi_2_O_3_:Ho^3+^ films were deposited on microscope glass at a temperature of 400 °C using the PLD technique. The Bi_2_O_2.3_ phase stronger at the higher O_2_ partial pressures, which indicated that oxygen vacancies occurred in the films. The surface morphologies of the 5 and 20 mTorr PO_2_ showed needle shape particles. The film deposited at an O_2_ partial pressure of 20 mTorr exhibited smoother surfaces, while the films deposited at lower and higher than the 20 mTorr PO_2_ displayed higher roughness values. The green and NIR emissions obtained from the film deposited at an O_2_ partial pressure of 20 mTorr showed an intensity that was more than 10 times higher than the film deposited in a vacuum. The best photocatalytic activity of the RhB degradation was attained for the films deposited at PO_2_ of 5 and 200 mTorr, owing to the modified surface morphologies and due to the high surface roughness. The PL and photocatalytic activities depend strongly on the surface morphology of the Bi_2_O_3_:Ho^3+^ films.

## Data availability

Data is not publicly available, but data will be made available on request.

## CRediT authorship contribution statement

**J. Divya:** Writing - original draft, Methodology, Investigation, Data curation, Conceptualization. **N.J. Shivaramu:** Writing - review & editing. **H.C. Swart:** Writing - review & editing, Supervision, Resources, Project administration, Methodology, Funding acquisition.

## Declaration of competing interest

The authors declare that they have no known competing financial interests or personal relationships that could have appeared to influence the work reported in this paper.
